# Trends in Insomnia, Burnout, and Functional Impairment among Health Care Providers over the First Year of the COVID-19 Pandemic

**DOI:** 10.2174/17450179-v18-e2206200

**Published:** 2022-07-15

**Authors:** Ahmed Yassin, Abdel-Hameed Al-Mistarehi, Aref A. Qarqash, Ola Soudah, Reema A. Karasneh, Sayer Al-Azzam, Aws G. Khasawneh, Khalid El-Salem, Khalid A. Kheirallah, Basheer Y. Khassawneh

**Affiliations:** 1 Department of Neurology, Faculty of Medicine, Jordan University of Science and Technology, Irbid, Jordan; 2 Department of Public Health and Family Medicine, Faculty of Medicine, Jordan University of Science and Technology, Irbid, Jordan; 3 Faculty of Medicine, Jordan University of Science and Technology, Irbid, Jordan; 4 Department of Basic Medical Sciences, Faculty of Medicine, Yarmouk University, Irbid, Jordan; 5 Department of Clinical Pharmacy, Jordan University of Science and Technology, Irbid, Jordan; 6 Department of Psychiatry, Faculty of Medicine, Jordan University of Science and Technology, Irbid, Jordan; 7 Department of Internal Medicine, Faculty of Medicine, Jordan University of Science and Technology, Irbid, Jordan

**Keywords:** COVID-19, Health care providers, Insomnia, Burnout, Functional impairment, One-year

## Abstract

**Background:**

COVID-19 pandemic has negatively impacted the psychological well-being and quality of life of health care providers (HCPs).

**Objectives:**

This study assessed the trends in prevalence and predictors of insomnia, burnout, and functional impairment among HCPs over the first year of the pandemic.

**Methods:**

An online survey was conducted one month after the pandemic’s onset (onset group) and a year later (one-year group). The demographic features of participants were collected. Insomnia, burnout, and functional impairment were assessed using Insomnia Severity Index (ISI), Mini-Z survey, and Sheehan Disability Scale (SDS), respectively.

**Results:**

The onset group included 211 HCPs (mean (SD) age 34.7 (9.3) years and 73% men), while 212 HCPs participated in the one-year survey (mean (SD) age 35.9 (10.5) years and 69% men). High prevalence estimates were found in both onset and one-year groups of symptoms of insomnia (52% *vs.* 49%), of diagnosis of clinical insomnia (15% *vs.* 18%), with a high mean ISI score (8.4 *vs.* 8.7), but with no significant difference between the onset and one-year groups. Risk factors for clinical insomnia included age in both groups, lower income and contact level with COVID-19 patients/samples in the onset group, and lower Mini-Z scores and higher SDS scores in the one-year group. Approximately one-third of respondents reported at least one or more burnout symptoms, with a higher percentage in the one-year group (35.4%) than in the onset group (24.2%) (*p=0.012*). Younger age, lower monthly income, and higher ISI and SDS scores were risk factors for burnout in both groups. Greater perceived changes in social life were associated with burnout in the onset group. In contrast, higher weekly working hours, worse participants’ evaluation of their institution’s preparation, and more changes in workload were risk factors for burnout in the one-year group. The SDS score and its subscales scores were higher in the one-year group than in the onset group. Changes in workload and social life predicted higher SDS scores among both groups. Living with older people predicted higher SDS scores among the onset group, while contact level and estimated number of COVID-19 patients that participants engaged in during caring predicted higher SDS scores among the one-year group. ISI scores were significantly correlated with the Mini-Z scores and SDS scores in both groups, while the Mini-Z and SDS scores were significantly correlated only in the one-year group.

**Conclusion:**

This study demonstrated high rates of insomnia, burnout, and functional impairment among HCPs during the pandemic. It reveals a significant rise in job burnout and functional impairment of HCPs overtime during the pandemic. Furthermore, high-risk subgroups are also highlighted for whom comprehensive psychosocial and occupational interventions might be warranted.

## 1 INTRODUCTION

For over two years, the world has been battling Severe Acute Respiratory Syndrome Coronavirus-2 (SARS-CoV-2). The effects of the Coronavirus Disease 2019 (COVID-19) pandemic have been vast, affecting different aspects of life [1-8]. By the end of February, 2022, more than 440 million cases and over 5.9 million deaths were reported worldwide [9]. The spectrum of clinical complications of COVID-19 is broad and includes cardiopulmonary, hemostatic, and neurologic effects [10-14]. In addition to the physical health sequelae, COVID-19 has deeply affected the psychological and mental health of patients and the public [15-17]. A survey conducted in the early stage of the COVID-19 pandemic in Sweden found that 22.2% and 28.3% of the population reported depressive and anxiety symptoms, respectively [16].

The psychological welfare of health care providers (HCPs), the most important key players in our battle against COVID-19, has been significantly impacted [18-23]. During the acute surge of the pandemic in London, between April and May, 2020, a total of 1,127 HCPs were evaluated for their psychological well-being [19]. The authors reported that most HCPs (84%) scored psychological distress levels above the general population mean score [19]. An American study on the general population found that HCPs were significantly associated with depressed mood, anxiety, and psychological distress than other sub-groups of the general population [22]. Another study from Saudi Arabia reported high percentages of anxiety, depression, sleep disturbances, and distress among HCPs, ranging between 27.9% and 68.5% [20]. Moreover, a systematic review and meta-analysis analyzed 14 cross-sectional relevant studies indexed during the COVID-19 pandemic up to June 23^rd^, 2020, and found that about one-quarter of health care workers (3,070 out of 14,173 participants, 21.7%) had clinically depressive symptoms [23]. Also, the authors reported significant associations of the depressive symptoms reported by HCWs during the pandemic with the female gender, being suspected/confirmed COVID-19, and having an infected family member or friend [23].

During the COVID-19 pandemic, HCPs have suffered significantly from unprecedented levels of stress, insomnia, job burnout, and functional impairment [24-28]. Such psychopathological sequelae of the COVID-19 pandemic represent a hazard to the well-being of the HCPs and their responsibilities toward their patients. Çelmeçe and Menekay found that stress, anxiety, and burnout of HCPs engaged in managing COVID-19 patients affected their quality of life [27]. Carmassi *et al*. reported an estimated prevalence of 31% of post-traumatic stress disorder (PTSD) among health care workers facing the COVID-19 outbreak acute phase in Italy during April and May, 2020 [28]. Moreover, those who reported PTSD with major depression (28.4%) showed high levels of burnout and functional impairment [28]. An earlier Italian study carried out between April 1^st^ and May 1^st^, 2020, on a larger sample size of HCPs (n=265), found that burnout was associated with anxiety and depressive symptoms [29]. These psychopathic symptoms among HCPs facing the COVID-19 pandemic could be attributed to their worries about getting infected, their fears of transmitting the virus to their family members and loved ones, increased workload, the negative emotions that progressively build up over time, and the prolonged periods of lockdown and social isolation [30-38]. This COVID-19 pandemic could be a lesson to learn the potential psychiatric challenges and public health issues of emerging pandemics in the future [39].

**Fig. (1):**
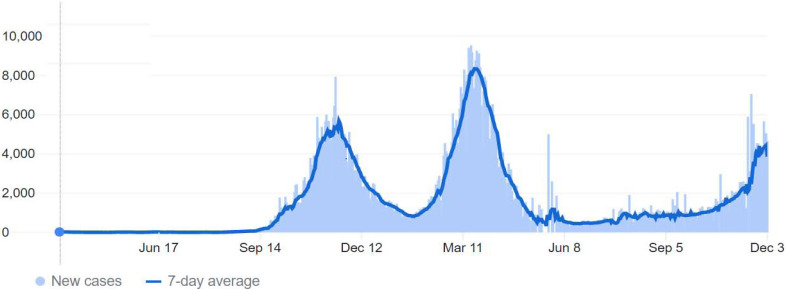
New daily and weekly Coronavirus Disease 2019 (COVID-19) cases in Jordan over 2020-2021. The first few cases were reported in March, 2020, with cases peaking for the first time in November, 2020 and then for a second time in April, 2021. The source of this figure is the COVID-19 dashboard of Johns Hopkins University’s center for systems science and engineering [9, 42].

The first few COVID-19 cases in Jordan were reported in March 2020, and cases rose in the subsequent weeks [11, 40]. Immediately, the government enforced a mass quarantine for three months, which limited the infection’s spread in Jordan. However, in the months following the lockdown ended, a significant rose in cases occurred as the “first peak”. Accordingly, the Jordanian authorities enforced incomplete restraints on people. These efforts resulted in a decline in the registered cases, and by the end of 2020, restraints were relaxed. After a few months, cases increased again, reaching the “second peak” around March, 2021 [9, 41, 42]. (Fig. **1**) demonstrates the COVID-19 pandemic progress in Jordan.

To examine how the influence of the pandemic on HCPs has changed over time, a multipurpose observational survey was distributed among a cohort of Jordanian HCPs one month after the pandemic’s onset and one-year later (in the second peak). We investigated the psychiatric measures of this impact by studying the change in anxiety symptoms among HCPs during the first year of the COVID-19 pandemic [43]. The other psychiatric measure that we focused on was depression, and thus we examined the change in its prevalence and predictors over time and reported the results in another paper [44].

The current study aims to look at the influence of the pandemic on the HCPs’ quality of life measures by investigating the trend in the prevalence and risk factors of insomnia, job burnout, and functional impairment over the first year of the pandemic. In addition, we assessed the potential correlations between these measures and how they affect each other in this context. The ultimate goal of this study is to shed light on the need for psychological interventions to alleviate the pandemic’s influence on the life quality of HCPs, particularly for high-risk groups. Thus, the findings of this study would be of interest to health care policymakers and administrators.

## 2 MATERIALS AND METHODS

### 2.1 Study Design, Population, and Ethical Considerations

This study had an observational cross-sectional design and was conducted using an anonymous electronic survey distributed among HCPs in two-time phases during the COVID-19 pandemic using the Google Form tool. In the first phase, it was disseminated one month after the pandemic’s onset in the country, between the 15^th^ and 30^th^ of April, 2020, and this group of HCPs was called the “onset group”. A year later, the same survey was disseminated, from the 15^th^ to the 30^th^ of March, 2021, and this group of respondents was called the “one-year group”. Although we targeted the same population in the two groups, it was not of the study criteria to involve the same subjects over the year of the COVID-19 pandemic. The eligible HCPs for participation in this study should be ≥18 years old, active workers during the COVID-19 pandemic, and living in Jordan. The participating HCPs in this study included those who work in a hospital or primary care institution as physicians, physician assistants, nurses, pharmacists, medical technicians, medical assistants, physical therapists, occupational therapists, psychologists, or performing services in allied human health professions. We used the snowball sampling method in distributing the survey, as the researchers sent out the survey through social media platforms and asked the participants to circulate it further to their colleagues. The questionnaire started with an informed consent page, including a brief description of the study goals and methodology, followed by an approval request for participation. If the person approves the consent, the questionnaire will proceed. Otherwise, it would terminate. No compensation was provided for participation in this study. The confidentiality of the participants’ answers and data was assured.

The Institutional Review Board (IRB) of the research and ethics committee at Jordan University of Science and Technology (JUST), Irbid, Jordan approved the study (IRB number: 106/132/2020). The study was also conducted following the Declaration of Helsinki. This work has been reported based on STOBE guidelines (Strengthening the Reporting of Observational Studies in Epidemiology) [45].

#### 2.1.1 Survey Instruments

The survey included previously validated structured scales to assess the possibility of insomnia, job burnout, job satisfaction, stress, and functional disability [46-52]. To assess the clarity of the questionnaire, it was first filled by twenty random HCPs, and subsequently, no major adjustments were needed. The survey had five sections: demographic features and COVID-19 personal history, occupation details, Insomnia Severity Index (ISI), Mini-Z survey, and Sheehan Disability Scale (SDS).

Participants first answered questions related to their demographics, including age, sex, residential area during the COVID-19 pandemic, marital status as currently married, widowed, divorced, or never married, the number of children they had, and if they had elderly (≥ 65 years) in their households. After that, questions about the personal history of COVID-19 were inquired, including whether the participant underwent COVID-19 testing or got infected by COVID-19 and, in case of infection, whether they were admitted to the hospital. Since the vaccine was unavailable when the questionnaire was distributed for the first time, the question about receiving the COVID-19 vaccine was included only in the one-year survey [53].

The second section included questions about occupation situation using questions asking about work position as a physician, nurse, pharmacist, technician, or other, number of years of experience, number of working hours per week, and monthly income in Jordanian Dinar (JD) (less than 500, 500 to1000, 1000 to 2000, or more than 2000). Then, the participants were asked whether they were directly contacting COVID-19 patients/samples and the estimated number of COVID-19 patients they dealt with (Zero, 1-49, 50-100, and >100). In addition, the participant’s assessment of their contact with COVID-19 patients or samples was evaluated using a five-point Likert scale, ranging from “1=lowest level” to “5=highest level”. The HCPs were asked whether they were provided any COVID-19-related education. The HCP’s assessment of their institutional readiness to manage COVID-19 patients was evaluated using a six-point Likert scale, ranging from “1=very bad” to “6=excellent”. Lastly, the impact of the pandemic on work and social life was investigated using two statements. The first one was the participant’s perception of the changes in workload and schedule during the pandemic with responses of “No perceived change / A little change / Some change / Much change / Very much change”. The other statement was how the HCPs perceive the levels of change in their social life using a four-point Likert scale, ranging from “1=No change” to “4=extreme change”.

##### 2.1.1.1 Insomnia Severity Index (ISI) Scale

The third section of the questionnaire included the ISI scale, a validated and reliable tool to quantify insomnia and its severity [46, 54-57]. This tool has already been successfully used to assess insomnia in remarkably large samples during the COVID-19 pandemic [46, 58]. The ISI scale consists of seven items covering the difficulties in initiating sleep, maintaining sleep, and awakening in the morning, as well as sleep pattern satisfaction, impairment due to sleep difficulties, distress level caused by the current sleep problems, and finally, the impact of these problems on the participant functioning during the day. Each item was rated based on the two-week interval before filling the survey using a five-point Likert scale, with zero indicating no problem and 4 indicating a very severe problem. After that, the total ISI score, ranging from 0 to 28, was calculated for each participant by summing the items’ scores. The higher ISI total scores indicate greater insomnia severity. Thus, the total score was interpreted as follows: no clinical insomnia (0-7), sub-threshold insomnia (8-14), moderate insomnia (15-21), and severe insomnia (22-28) [56]. Also, the participants with a total ISI score of ≥15 (moderate and severe) were at high risk of clinical insomnia, and thus, binary classification of participants was conducted by a cutoff of 15 [56, 57]. Cronbach’s alfa (α) of the items of ISI was 0.906, indicating excellent internal consistency and reliability.

##### 2.1.1.2 Mini-Z Survey

The fourth section of the e-survey included the most recent version of the Mini-Z survey (version 2.0) [48, 49, 59]. The Mini-Z scale is a brief tool consisting of ten items measuring burnout, its potential risk factors, job satisfaction, and stress. Burnout measurement was based on a single question asking the participants to classify their level of burnout using their definitions with five choices, including:

1. I enjoy my work. No symptoms of burnout. (Score 1).

2. I am under stress and do not always have as much energy as I did, but I do not feel burned out. (Score 2).

3. I am definitely burning out and have one or more symptoms of burnout, *e.g*., emotional exhaustion. (Score 3).

4. The symptoms of burnout that I am experiencing will not go away. I think about work frustrations a lot. (Score 4).

5. I feel completely burned out, and I am at the point where I may need to seek help. (Score 5).

This single-item burnout measurement was adapted from the Physician Worklife Study and validated against the Maslach Burnout Inventory’s emotional exhaustion scale in several studies [59-61]. The high risk of burnout was defined by scoring ≥3 on the burnout measurement item of the Mini-Z survey [49, 52].

The Mini-Z survey also includes seven items measuring potential burnout drivers, including alignment of participant’s values with those of clinical leaders, teamwork assessment, work control, documentation time sufficiency, Electronic Medical Record (EMR) work at home, Electronic Health Record (experience) proficiency, and clinic work area chaos (pace). Besides, two items assessing job satisfaction and stress feelings are included in the Mini-Z survey. Response to each item of the Mini-Z survey ranges from 1 to 5, with 5 being the most satisfactory and positive response. Thus, the previously mentioned scores for the burnout measurement item will be reversed. A summary score of 10-50 is obtained for each participant by summing the scores of the 10 Mini-Z items. A summary score of ≥40 defines a “joyful workplace”. The Mini-Z survey is further divided into two 5-item subscales, and each one has a total score ranging from 5 to 25 [48]. “A highly supportive work environment practice” is represented by the subscale score of ≥ 20. “A reasonable work pace and manageable EMR stress” is defined by the subscale score of ≥ 20 [48]. The Cronbach’s α of the Mini-Z survey items was 0.719, indicating acceptable reliability.

##### 2.1.1.3 Sheehan Disability Scale (SDS)

Lastly, the participants’ functional impairment and disability levels were assessed using the SDS [50, 51, 62-64]. The SDS is a brief three-item self-reported tool that measures function impairment in 3 interrelated domains: work, social life/leisure activities, and family life/home responsibilities. Each domain is assessed using a ten-point visual analog scale (VAS), ranging from 0 to 10: 0 (Not disability at all), 1-3 (mild), 4-6 (moderate), 7-9 (marked), and 10 (extreme) disability. The scores of the three domains are added up to evaluate global functional impairment, with a total score ranging from zero (unimpaired) to 30 (highly impaired). The Cronbach’s α of SDS items was 0.759, signifying acceptable internal consistency and reliability.

### 2.2 Data Analysis

The Windows IBM Statistical Package for theire Social Sciences (SPSS) software, version 26.0, was used for analyzing the data. After verifying the normality of the dataset using both Q-Q plots and the Shapiro-Wilk test, the continuous variables were presented as mean ± standard deviation (m ± SD). The categorical variables were presented as percentages and frequencies. The age was presented as a categorical variable based on the participants’ age interquartile ranges, as 23 to 27, 28 to 31, 32 to 39, and 40 years or more. The number of children in the family for married participants was also categorized based on its interquartile ranges. The weekly working hours were further divided into two groups according to the participants’ median working hours of 40 per week. The Cronbach’s α was used for the three scales to measure the internal consistency reliability.

Univariate analyses were conducted to assess the differences between the onset and the one-year groups. Chi-square test or Fisher’s exact test was used for categorical variables, while student’s t-tests or one-way ANOVA were used for continuous variables. Pearson correlation, with its coefficient (r), was used to examine a possible linear relationship between the total mean scores of the three scales. The sample effect size was investigated using Phi (φ) for categorical data, considering 0.10 – <0.30 as a small effect, 0.30 – <0.50 as a medium effect, and ≥0.50 as a large effect for Phi (φ) statistics. Cohen’s d statistics was used to assess the effect size for student T-test and one-way ANOVA, considering 0.20 – <0.50 as a small effect, 0.50 – <0.80 as a medium effect, and ≥0.80 as a large effect [65, 66].

The binary logistic regression analyses were conducted to determine the potential risk factors for clinical insomnia and burnout separately among the onset and one-year groups of HCPs. In the clinical insomnia model, the dependent outcome variable was the high potential clinical insomnia diagnosis identified by a total score of ≥15 on the ISI scale, and thus, it included moderate and severe insomnia categories. While scoring ≥3 on the burnout measurement item of the Mini-Z survey, which indicates a participant with a high risk of burnout, was the dependent outcome variable in the burnout model.

The stepwise backward approach with a cutoff p-value of *0.2* was applied to select the most parsimonious model for identifying the potential risk factors among the independent explanatory variables in each model. The independent explanatory variables were age groups, gender, marital status, occupation, living with the elderly, weekly working hours categories, monthly income classifications, history of COVID-19 testing or infection, the participants’ assessment of their contacts with infected patients, getting COVID-19 related education, the participants’ evaluations of their institution’s readiness to deal with COVID-19 patients, their perception about workload and schedule changes, and their perceived levels of change in their social life. Also, the total scores of the Mini-Z survey and SDS were included as independent explanatory variables in the clinical insomnia model, while the total scores of ISI and SDS were included as independent explanatory variables in the burnout model. The explanatory variables in the last model were checked for multicollinearity using the variance inflation factor (VIF). The adjusted Odds Ratio (OR) and 95% Confidence Interval (95% CI) were reported. A cutoff-point p-value of *≤ 0.05* was considered for statistical significance.

## 3 RESULTS

### 3.1 Participants’ Baseline Characteristics

A total of 211 HCPs participated in the first round of the study and represented the onset group, while 212 participated in the second round a year later and represented the one-year group. The mean (SD) age of participants was 34.7 (9.3) within the onset group and 35.9 (10.5) years for the one-year group, with a male predominance in both groups (73% and 69%, respectively). Most of the participants in both groups were married (63% and 58%, respectively), reported lower than one-thousand JD monthly income (68% and 55%, respectively), were physicians (78% and 85%, respectively), with resident physicians constituting the most considerable portion (46% and 46%, respectively).

Regarding the COVID-19 vulnerability, the one-year group had a higher level of contact with COVID-19 than their onset group counterparts, with the score “5” reported by 26% and the score “4” reported by 30% of the one-year group compared to 7% and 11% of the onset group, respectively. The differences in contact levels with COVID-19 between the groups were statistically significant (*p<0.001*). Furthermore, a higher proportion of participants from the one-year group reported direct contact with COVID-19 patients or samples (68%) than among participants of the onset group (23%) (*p<0.001*). This finding was supported by a higher estimated number of patients that the one-year HCPs dealt with than the onset group (*p <0.001*). The one-year sample also had higher proportions of participants who underwent testing (89%) and got infected (42%) than onset group participants (22%, Zero; respectively) (*p<0.001 for each*). Lastly, the one-year group participants reported a statistically significant worse assessment of the institutional readiness to care for COVID-19 patients than the onset group (*p =0.003*), with a higher proportion in the worst categories; “Very bad” (5% *vs.* 2%, respectively), “Bad” (17% *vs.* 10%, respectively), and “Fair” (31% *vs.* 20%, respectively).

Regarding work and social life changes, most of the participants reported “Very much change” and “Much change” in work schedule and intensity (Onset group: 69%; One-year group: 65%). Also, most of the participants reported “Extreme changes (5)” and “4” in social life to avoid transmitting the disease to loved ones (Onset group: 79%; One-year group: 80%). However, both changes were not statistically significant between the two groups (*p=0.378* and *p=0.782*, respectively). Comparative socio-demographics, occupational characteristics, COVID-19 vulnerability, and work and social life changes between the two groups are presented in Table **1**.

#### 3.1.1 Differences in Scales’ Findings between the two Groups’ Participants

The mean (SD) score of ISI was 8.7 (6.3) in the one-year group, which was marginally higher than the onset group (M=8.4, SD=5.8). This difference, however, was not statistically significant (*p=0.655*) and was of trivial effect with d=0.05 (95% CI: -0.14 – 0.24). A similar pattern was observed in the insomnia severity categories between the two groups. Comparisons of the scales’ results and their variations are presented in detail in Table **2**. The Mini-Z scale had insignificant results, with the onset group having a slightly higher mean (SD) summary score of 32.2 (5.6) than the one-year group (31.1 (5.8)), with a marginally small effect with a p-value of *0.061* and d=0.19 (95% CI: 0.0 – 0.38). Also, the proportions of participants having a “Joyful Workplace” were not statistically different between the onset group (9%) and the one-year group (6%) (*p=0.250*). Similar insignificant differences were observed between the two groups in the mean (SD) scores of the supportive work environment practice subscale (16.6 (3.2) *vs.* 16.1 (3.3), *p=0.087*) and the work pace and EMR stress subscale (15.5 (3.1) *vs.* 15.0 (3.2), *p=0.104*). Also, insignificant differences in the proportions between the two groups were observed in categorical variations of the Mini-Z survey subscales. On the other hand, the one-year participants had a significantly higher mean (SD) total score of the SDS (M=22.8, SD=5.3) than the onset ones (M=20.9, SD=6.0) (*p<0.001*), but this difference was of a small effect, d=0.34 (95% CI: 0.14 – 0.53). Similar significant higher scores were observed across SDS subscales, including disrupted work, disrupted social life and leisure activities, and disrupted family life and home responsibilities (Table **2**).

#### 3.1.2 Correlations between the Scales

The possible linear correlations between the different scales for each group are presented in Table **3**. The ISI and Mini-Z survey total scores had a significant negative correlation in each participants’ group (Onset group: r = -0.260, *p<0.001*; One-year group: r = -0.370; *p<0.001*). The insomnia score was also significantly but positively associated with the SDS score in both groups (r = 0.192, *p=0.005;* and r = 0.343, *p=<0.001* for the onset and one-year groups, respectively). Lastly, the scores of the Mini-Z survey and SDS were negatively and significantly associated in the one-year group (r = -0.288, *p<0.001*), while there was almost no correlation observed in the onset group (r = 0.006; *p=0.932*).

#### 3.1.3 The Differences in the Mean Scores of Scales’ by the Characteristics of Participants in the Onset Group

Among the onset group participants, significantly higher ISI mean scores were observed among participants with a lower number of children; as those who had one or two children reported the highest mean (SD) ISI score (9.84 (6.22)), while those with more than four having the lowest mean (SD) ISI score (6.44 (4.69)) (*p=0.019*). Also, the participants with lower than 1000 JD monthly income had a mean (SD) ISI score of 9.1 (6.0), which was significantly higher than their counterparts (6.8 (4.8)) (*p=0.007*). The ISI scores significantly differed according to how the participants evaluated the readiness of the institution to manage COVID-19 patients in the onset sample (*p=0.043*). Those with a “Very bad” evaluation had the highest ISI score (M=10.8, SD=8.1), while their “Excellent” counterparts reported the lowest score (M=4.3, SD=4.6). On the other hand, the ISI scores were not significantly different by the onset group participants’ demographic and occupational characteristics of age, sex, marital status, occupation, living with older people, and working hours (*p>0.05*). Also, testing, contact levels with COVID-19, lack of special education, and perception about work and social life changes had no association with ISI scores in the onset sample (*p>0.05*). The mean scores differences across the participants' characteristics of the onset group are presented in Table **4**.

The Mini-Z survey had somehow similar scores across the first three age groups, with a mean (SD) score of 31.2 (5.3) among those aged <40 years, while those who were 40 years or older had a higher mean (SD) score (35.3 (5.3)) (*p<0.001*). The data from this sample demonstrated a trend of a significant increase in the Mini-Z survey summary scores with the increasing number of children (*p=0.008*), increasing monthly income (*p<0.001*), better participants’ evaluation of their institutions’ preparedness (*p=0.001*), and more remarkable changes in social life (*p=0.004*). The Mini-Z summary scores were also significantly associated with the contact level assessment (*p=0.001*), but no clear linear relationship was reported (Table **4**).

For the SDS scores among the onset group participants, HCPs who perceived extreme changes in work or social life had the highest SDS mean scores compared to those who perceived no or minor changes (*p=0.036, p<0.001*; respectively). Unlike ISI and Mini-Z survey, the mean (SD) score of SDS was significantly higher among participants who lived with the elderly than those who did not (22.0 (5.6) *vs.* 19.9 (6.1), *p=0.010*).

In the onset group, the scores of the three scales did not differ by the participants’ gender, marital status, occupation, working hours per week, COVID-19 testing history, and lack of special COVID-19 education.

#### 3.1.4 The Differences in Scales’ Scores by the Participants’ Characteristics in the One-Year Group

Table **5** summarizes the scores of the ISI, Mini-Z survey, and SDS across the participants’ characteristics of the one-year group. Similar to the onset group findings of ISI scores, one-year participants who had lower income and low satisfaction with the institution’s readiness had higher ISI scores. However, extreme changes in work or social life in the one-year group were associated with the highest insomnia mean scores compared to those perceived no or minor changes (*p<0.001*, *p=0.003*; respectively). Unlike the onset group, no significant association was observed between the ISI scores and children number and participants’ evaluation of the institution’s preparedness among one-year participants.

Among the one-year participants, higher Mini-Z summary scores were observed with the older ages (*p=0.023*), married participants (*p=0.021*), other than physician occupation (*p=0.050*), and working for less than 40 hours weekly (*p=0.001*) than their counterparts. This scale also had an increasing pattern with the higher monthly income (*p=0.004*), lower contact level with the infection (*p<0.001*), fewer COVID-19 cases that HCPs contacted (*p=0.015*), having a better evaluation of the institution’s preparedness (*p<0.001*), and lastly perceiving no or little work schedule and intensity changes (*p=0.009*). On the other hand, the mean (SD) of the Mini-Z summary score was significantly lower among one-year participants who had undergone testing (30.8 (5.7)) than those who had not (33.5 (5.7)) (*p=0.035*). However, there was no association between the history of COVID-19 infection and the Mini-Z summary score in the one-year group (*p>0.05*).

In the one-year group, trends of increasing SDS scores were observed with more perceived changes in workload (*p=0.041*) and social life (*p<0.001*). These findings are similar to what was previously observed in the onset sample. However, unlike the onset group, no significant difference was observed in the mean (SD) of SDS scores between one-year HCPs who reported living with the elderly (22.7 (5.6)) and those who did not (22.8 (4.8)) (*p=0.887*). Positive associations were noticed between the SDS scores and COVID-19 contact level (*p=0.003*) and the estimated number of COVID-19 patients that participants dealt with (*p=0.018*) (Table **5**).

The one-year participants’ gender, children number, living with elderly, history of COVID-19 infection, and lack of COVID-19-related education were insignificantly different in the scores of ISI, Mini-Z survey, and SDS.

#### 3.1.5 Prevalence Estimates of and Risk Factors for Clinical Insomnia among HCPs

A total of 70 (16.5%) participants were at high risk for clinical insomnia, including 31 (15%) in the onset group and 39 (18%) within the one-year sample. Binary logistic regression analyses, presented with odds ratios in Table **6**, showed that 40 years of age or older and having lower monthly income and COVID-19 contact level were independent risk factors for clinical insomnia in the onset group. While being in middle-aged groups (28-39 years old), having higher SDS scores and lower Mini-Z summary scores were the independent risk factors for developing clinical insomnia in the one-year group.

#### 3.1.6 Prevalence and Risk Factors for Burnout in HCPs

Among responders, about half (n=181, 42.8%) experienced stress but no burnout, while 116 (27.4%) reported enjoying their work. Persistent symptoms of burnout were reported by 25 (5.9%) responders, and ten responders (2.4%) reported being completely burned out. Fig. (**2**) demonstrates the distribution of burnout symptoms among participants. Approximately one-third (n=126, 29.8%) of participants reported at least one or more burnout symptoms, with ≥3 scoring on the burnout measurement item of the Mini-Z survey. Of the one-year group, a higher proportion (n=75, 35.4%) was at high risk of burnout than the onset group (n=51, 24.2%) (*p=0.012*).

Common risk factors for burnout between the two groups included younger age, lower monthly income, and higher ISI and SDS scores. Greater perceived changes in social life due to the pandemic predicted burnout in the onset sample. On the other hand, the risk factors for burnout in the one-year sample included HCPs with higher weekly working hours, who had a worse impression of their institution’s preparedness toward COVID-19, and those with significant perceived changes in their workload and schedule during the COVID-19 pandemic (Table **7**).

## 4 DISCUSSION

This study is one of the first surveys that gain insights into the changes in the prevalence estimates and predictors of insomnia, burnout, and functional impairment among HCPs over the first year of the COVID-19 pandemic. The study showed that HCPs have high prevalence estimates of insomnia, job burnout, and functional impairment during the pandemic. Of notice, job burnout and functional impairment have significantly increased over a year of the pandemic. Insomnia, however, has not. The risk factors for these quality-of-life measures significantly differed by socio-demographic characteristics and occupation features, with variations in these factors observed over the first year of the pandemic. Clinical insomnia in the onset group was associated with lower monthly income and contact levels with COVID-19 patients and samples. In contrast, after one year of the pandemic onset, clinical insomnia symptoms increased among HCPs with lower Mini-Z scores and higher SDS scores. Age was a constant risk factor in both groups. Higher perceived social life change was a predictor for burnout in the onset sample, while higher weekly working hours, worse evaluation of the institution’s preparedness, and perceiving more significant changes in workload were predictors in the one-year sample. However, younger age, lower monthly income, and higher ISI and SDS scores continued to be predictors of burnout over the year. As for functional impairment, living with the elderly was correlated with higher scores on the SDS score among the onset sample, while contact level and estimated number of COVID-19 patients that HCPs dealt with were predictors of higher SDS scores among the one-year participants. Perceived remarkable changes in workload and social life had significant associations with higher SDS scores in both groups. The majority of these scales were significantly correlated with each other.

### 4.1 Insomnia

A cross-sectional survey from China on 7,236 participants found that HCPs were more likely to report poor sleep quality than other occupation participants [67]. A cross-sectional study conducted from October to November, 2020, in Dhaka, Bangladesh, on a total of 586 adult participants found that the prevalence estimate of clinical insomnia (ISI ≥15) among adults during the COVID-19 pandemic was 12.7% [68]. Compared with the general population in the latter study, HCPs in our study had a higher percentage of clinical insomnia (15% in the onset sample and 18% in the one-year sample). A meta-analysis with 33,062 HCPs across five studies reported 38.9% as the insomnia prevalence estimate [69]. A cross-sectional study on 1,257 HCPs from 34 hospitals in China found that 34.0% reported insomnia symptoms, and 7.8% had clinical insomnia [24]. Compared with these studies on HCPs, our study showed a higher rate of insomnia symptoms (52% in the onset sample and 49% in the one-year sample) and clinical insomnia (15% in the onset sample and 18% in the one-year sample) among HCPs. A Spanish study on HCPs found that the mean (± SD) ISI score was 7.83 ± 5.29 [70]. However, the HCPs in our cohort reported higher mean (± SD) ISI scores at the beginning of the COVID-19 pandemic (8.4 ± 5.8) and after one year (8.7 ± 6.3).

A cross-sectional study from China reported that females, frontline workers, nurses, and those in Wuhan, where the COVID-19 pandemic started, experienced more severe symptoms of depression, anxiety, insomnia, and distress [24]. The engagement of HCPs in caring for COVID-19 patients for prolonged hours and the dramatic changes in their workload and schedule have been linked to psychological distress, resulting in poor sleep quality and insomnia [71]. Our study found that the perceived contact level with COVID-19 patients was an independent significant risk factor for clinical insomnia in the onset group.

A prospective cohort study comprising 2,089 HCPs from Spain found that older professionals (>55 years) reported lower rates of insomnia [72]. However, in our study, older age was a risk factor for clinical insomnia in both samples. In our study, less monthly income was significantly associated with insomnia symptoms in the onset sample. The unpredictability effects of the pandemic on the economic status of HCPs at the pandemic onset might have added to their worry, making this factor significantly associated with clinical insomnia in the onset sample. Many studies emphasized the harmful effects of low financial status on the psychological well-being of adults during the hard times of the pandemic [3, 16, 73, 74]. Instead, financial support by the government was shown to mitigate these detrimental psychological consequences [75].

The study findings indicated that elderly HCPs, those with low monthly income, and who had the possibility of contact with a patient with COVID-19 in the workplace are most in need of tailored mental and occupational health interventions to be protected from insomnia development. Our findings reported that lower Mini-Z summary scores and higher SDS scores were independent risk factors for clinical insomnia in the one-year group only, demonstrating the negative influence of the pandemic’s chronicity on HCPs’ well-being, resulting in job stress, burnout, dissatisfaction, disability, and functional impairment on the individual quality of sleep. Thus, interventions for insomnia are needed to target different socio-psychological factors.

### 4.2 Job Burnout

A significant proportion of our HCPs, *i.e*., one-third, reported at least one or more symptoms of burnout, with a significant rise of 11.2% in the one-year sample compared to the onset sample. A cross-sectional survey involving 605 HCPs in Baltimore, USA, found that HCPs involved in the management of COVID-19 patients were more likely to have higher burnout scores than HCPs who were not in direct contact with such patients [76].

Younger HCPs in our study had a higher burnout risk than older participants in both groups. Similar to our finding, a previous study from London reported higher psychological distress and burnout among the younger HCPs [19]. Other variables significantly associated with burnout among both groups included lower monthly income and higher ISI and SDS scores.

In the onset group, perceiving more remarkable social changes due to the pandemic was a risk factor for burnout. Social life during the first survey was limited due to the lockdown, which could have increased distress and negatively impacted sleep. Literature showed higher psychological distress and depressive symptoms in those living alone or socially isolated [77-79].

Teo *et al*. (2021) conducted a six-month multicenter prospective study on 2,744 HCPs in Singapore to measure worker anxiety, stress, and burnout during the pandemic [25]. The authors found that 24% of HCPs had elevated perceived job burnout, which increased by 1.0% and 1.2% monthly [25]. Similar to our study’s findings on the one-year group, prolonged working hours in the Teo *et al*. study were a risk factor for burnout [25]. Also, it was suggested that increasing workload during the COVID-19 pandemic was directly correlated with increased burnout [80]. In line with such findings from the literature, our HCPs who reported significant burnout risk within the one-year sample included those who had more changes in workload and had prolonged weekly working hours. Worse participants’ impression about their institution’s readiness to manage COVID-19 cases, which might have been affected by the progressive increase in the pandemic burden over time, was significantly associated with burnout among the one-year group. Thus, the significant increasing trend of burnout after one year of the pandemic could also be explained by the higher workload burden on the HCPs, increased responsibilities of providing patients and families with regular updates, and their feelings of helplessness for inability to provide the patients enough help or support with the tremendous increase in COVID-19 cases, such as in light of reported lack sufficient knowledge about patient confidentiality and data sharing among Jordanian HCPs [81].

The study findings necessitate the need for interventions to alleviate the COVID-19 pandemic contribution to the HCPs’ burnout, including improvement of the occupational environment by balancing the workload on HCPs and their income, modulating working hours accordingly, providing employees with a flexible schedule, considering their feedback regarding the institutional policies, and addressing their psychological needs in light of COVID-19 related social changes. Others also suggested more measures to alleviate the COVID-19-related burnout among HCPs, including exercise, a balanced diet, better sleep pattern, social support, and occupational environment enhancement by avoiding blame, sharing experience and advice, sharing management decisions, forming psychosocial support teams, and involvement of HCPs in developing strategies to decrease burnout [82-85]. Better job-person fit, appreciation at work, and congruent worker-organization goals and values were associated with lower burnout among HCPs [86]. These tailored mental and occupational health interventions should target the high-risk groups for burnout, such as young HCPs, those with low monthly income, who have remarkable social and workload changes due to the pandemic, and who work for prolonged hours. Regular assessments of the burnout among HCPs and identifying burnout drivers related to effective organizational structure and supportive teamwork in practice personnel are recommended.

### 4.3 Functional Impairment/disability

A study on 102 nurses from Malawi was conducted between August and September, 2020, using the Work and Social Adjustment Scale (WSAS) and found that 48% of nurses had COVID-19-related functional impairment [87]. A study on 389 Malaysian HCPs found that their social relationship QoL was lower than usual, with COVID-19-related challenges, such as interrupted daily routine, heavy exposure to COVID-19 patients, and psychological effects, including depression, anxiety, and stress were significant predictors for lower QoL among HCPs [26]. These findings were congruent with our finding that perceiving more remarkable changes in workload and social life were linked to higher SDS scores among both groups. In addition, contact level and estimated number of COVID-19 patients that HCPs dealt with, which have progressively increased over time, had a significant association with higher SDS scores in the one-year sample. This also explains the increase in functional impairment scores over the first year of the pandemic, which emphasizes the role of stress chronicity in contributing to the global dysfunctionality of HCPs.

These findings shed light on the importance for hospital managers and policy makers to carry out strategies that promote job control, provide employees with job resources, and reduce workers' workload to reduce the functional impairment risk. Health care managers may improve workers' sense of control by promoting their autonomy in the workplace. In fact, job autonomy is considered an essential coping strategy for decreasing job strain [88]. Our study also demonstrates that living with the elderly correlated with higher SDS scores among the onset sample. This finding indicated the reciprocal effects of home and work environments, and it could be explained by the amplified worry of HCPs at the pandemic’s onset about the potential of transmitting the infection to their old parents and loved ones, knowing the worse outcome of COVID-19 in the elderly [89].

### 4.4 Correlations between the Different Scales

The ISI and Mini-Z summary scores had a significant negative correlation in both groups, and the ISI score was also significantly associated with the SDS score but with a positive direction in both groups. In contrast, the burnout and SDS scores were negatively and significantly associated in the one-year group only. These findings indicated the reciprocal effects between these measures and stress, thus indicating the need for a holistic approach to improving the quality of HCPs’ lives, including improving their sleep, work environment, and family and social support systems [82, 86]. Also, these correlations indicated the need for addressing different and multiple psychological effects of the pandemic on HCWs rather than focusing on a solo aspect. Interventional programs should be aimed at reducing HCPs’ experience of stressors and different socio-psychological factors and, subsequently, should be directed toward both individuals and organizations.

### 4.5 Study Limitations

This study is conducted in a single center and has a small sample size. The observational cross-sectional survey used in the study has its fundamental shortcomings. The study did not survey the same respondents to find the actual trend of changes in their insomnia, burnout, and functional impairment. Thus, this study did not investigate the longitudinal associations, and the potential overlap between subjects of the onset group and those belonging to the one-year group cannot be excluded. However, this limitation was relatively offset by targeting the same population and applying the same inclusion and exclusion criteria in the two-time phases of the study. Moreover, the results indicated a match with insignificant statistical differences between the onset and one-year samples in their socio-demographic characteristics.

Using a snowball sampling approach in distributing the survey and data collection is another limitation of this study, with its associated sampling bias and margin of error. Snowball sampling, like any non-random sampling method, does not guarantee the representation of the participants, and there is no way of knowing how precisely the participants meet the study participation criteria since this responsibility is in the hands of the participants themselves. Additionally, because this is an online survey-based study, the results are subject to recall, and we could not check if participants’ responses were accurate. However, previous studies have shown that online-based snowball survey is a cost-effective method that can reach effectively targeted people otherwise unreachable, especially in the context of the COVID-19 pandemic, provides easier access to the survey, and provides a private and safe environment for the respondents to answer questions honestly and accurately compared to face-to-face interviews [90-92]. Thus, we recommend conducting a snowball sampling recruitment method by inserting an instructional manipulation check, such as a blue-dot task, to increase the statistical power and reduce the signal-to-noise ratio, as well as achieving a larger sample of respondents by providing appropriate incentives is suggested [93-95].

Of notice, physicians and males represented most of the participants. Although this was relatively similar in both samples, generalizing the study’s findings to all HCPs, particularly female HCPs, is less precise. However, this can partially be justified by the statistical fact that most (70%) of the HCPs in Jordan are males [96]. Also, this study did not investigate the potential mechanisms for coping with reported insomnia, job burnout, and functional impairment among HCWs and the potential effects of exercise, dietary habits, nicotine dependence, smoking, comorbidities, disabilities, and laboratory investigations [15-97, 106-109]. Thus, prospective studies with a larger sample size examined these potential factors for insomnia, job burnout, and functional impairment and investigated potential protective interventions.

## CONCLUSION

The COVID-19 pandemic has significantly impacted the quality of life among HCPs. This study demonstrated high prevalence estimates of insomnia, burnout, and functional impairment among HCPs. Job burnout and functional impairment have significantly increased over the first year of the pandemic. This necessitates regular assessments of insomnia, burnout, and functional impairment among HCPs, thus applying swift interventions to address the mental health needs of HCPs. The associations of socio-demographic and occupational characteristics with insomnia, burnout, and functional impairment were evident among HCPs in our study, but these associations changed over time. Thus, identifying insomnia, burnout, and functional impairment drivers and subsequently targeting vulnerable groups considering the socio-demographics and work environment factors at play in this situation should be used to fine-tune the interventions. The related factors included age, income, workload, work schedule, contact levels with patients, and changes in social life. Mental and occupational health interventions are needed to target these factors. Since the quality of life measures correlate and could affect each other, a comprehensive approach to mitigating the harmful influence of the pandemic could not be stressed enough. Thus, health-providing institutions that identify well-being as a quality system of measurement can begin the journey to assessing and reducing the levels of insomnia, burnout, and functional impairment among their employees, thus, hopefully improving the quality of life and satisfaction of their health care workforce and the quality of care for their patients. Psychosocial interventions are needed to help HCPs better respond to COVID-19 and future pandemics.

## Figures and Tables

**Fig. (2):**
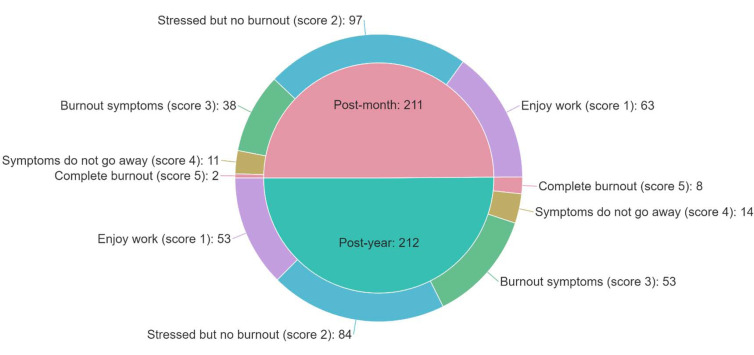
Prevalence estimates of the levels of burnout symptoms as self-defined by the participants, displayed as counts.

**Table 1:** Social, demographic, and occupational features; COVID-19 vulnerability; and perceived alterations in workload and social life of the participating health care providers.

**Variable**		**Onset group, n=211** **n (%)**	**One-year group, n=212** **n (%)**	***p-value***
**Socio-Demographic Characteristics**				
**Age, y**	Mean ± SD	34.7 ± 9.3	35.9 ± 10.5	0.241
	Min-Max	24-70	23-73	
**Gender**	Male	154 (73)	147 (69)	0.408
	Female	57 (27)	65 (31)	
**Marital status **(*)	Never married	79 (37)	89 (42)	0.340
	Married	132 (63)	123 (58)	
**Number of children in the family (among married ones)**	Zero	13 (10)	11 (9)	0.901
	1-2	56 (42)	58 (47)	
	3-4	47 (36)	40 (33)	
	>4	16 (12)	14 (11)	
**Living with the elderly (65 years or older)**	Yes	93 (44)	122 (58)	** 0.006**
**Occupational features**				
**Occupation (†)**	Physician	164 (78)	180 (85)	0.058
	Others	47 (22)	32 (15)	
**Experience years**	Mean ± SD	9.5 ± 8.9	10.6 ± 10.3	0.253
**Working hours per week**	Mean ± SD	43.6 ± 17	44.6 ± 18.9	0.599
	<40 hours	43 (20.4)	59 (27.8)	0.093
	≥40 hours	169 (79.6)	153 (72.2)	
**Monthly income (in JDs)**	<500	24 (11)	21 (10)	** 0.026**
	500-1000	120 (57)	95 (45)	
	1000-2000	26 (12)	30 (14)	
	>2000	41 (19)	66 (31)	
**COVID-19 vulnerability**				
**Vaccination status (**¥)	Vaccinated	0 (0)	152 (72)	***-***
	Not vaccinated	0 (0)	60 (28)	
	Vaccine not available	211 (100)	0 (0)	
**Underwent COVID-19 testing**	Yes	47 (22)	189 (89)	** <0.001**
**Being in direct contact with COVID-19 patients or samples**	Yes	48 (23)	144 (68)	** <0.001**
**Estimated number of COVID-19 patients that health care providers dealt with**	Zero	165 (78)	73 (34)	** <0.001**
	1-49	34 (16)	67 (32)	
	50-100	8 (4)	30 (14)	
	>100	4 (2)	42 (20)	
**Perceived level of contact with COVID-19 patients**	1 (Lowest)	49 (23)	13 (6)	** <0.001**
	2	62 (29)	22 (10)	
	3	62 (29)	59 (28)	
	4	23 (11)	63 (30)	
	5 (Highest)	15 (7)	55 (26)	
**Receiving a special education to deal with COVID-19 patients**	Yes	73 (35)	85 (40)	0.243
**Preparedness of institution to deal with COVID-19 patients**	Excellent	11 (5)	6 (3)	** 0.003**
	Very good	58 (28)	37 (18)	
	Good	74 (35)	59 (28)	
	Fair	42 (20)	65 (31)	
	Bad	21 (10)	35 (17)	
	Very bad	5 (2)	10 (5)	
**Got infected**	Yes	0 (0)	89 (42)	** <0.001**
**Hospital admission if infected**	Yes	-	3 (3)	*-*
**Perceived levels of change in work and social life due to the COVID-19 pandemic**				
**Change in work schedule and intensity**	No change	10 (5)	16 (8)	0.378
	Little change	17 (8)	13 (6)	
	Some change	39 (19)	45 (21)	
	Much change	75 (36)	82 (39)	
	Very much change	70 (33)	56 (26)	
**Change in social life**	1 (No change)	11 (6)	6 (3)	0.647
	2	33 (16)	36 (17)	
	3	87 (41)	87 (41)	
	4 (Extreme change)	80 (38)	83 (39)	

(*) The married category included currently married, divorced, and widowed participants.

(†) Others category in the occupation included pharmacists, nurses, and technicians.

(¥) The vaccine was unavailable in Jordan until January 2021.

**Table 2:** Insomnia severity index, mini-z survey, and sheehan disability scale comparisons.

**Scale**		**Onset Group, n=211** **n (%)**	**One-Year Group, n=212** **n (%)**	***p-Value***	**Effect Size (95%CI)**
**Insomnia Severity Index (ISI)**					
**Total ISI score**	Mean ± SD	8.4 ± 5.8	8.7 ± 6.3	0.655	0.05 (-0.14 – 0.24)
No clinical Insomnia	0-7	99 (47)	107 (50)	0.179	0.11 (-0.05 – 0.21)
Subthreshold Insomnia	8-14	81 (38)	66 (31)		
Moderate clinical Insomnia	15-21	28 (13)	30 (14)		
Severe clinical insomnia	22-28	3 (1)	9 (4)		
**Clinical insomnia (ISI score ≥15)**	Yes	31 (15)	39 (18)	0.305	0.05 (-0.05 – 0.15)
**Mini-Z Survey**					
**Summary score**	Mean ± SD	32.2 ± 5.6	31.1 ± 5.8	0.061	0.19 (0.0 – 0.38)
Joyful Workplace	Yes (≥40)	18 (9)	12 (6)	0.250	0.06 (-0.04 – 0.15)
**Supportive work environment practice subscale (Q1-Q5)**	Mean ± SD	16.6 ± 3.2	16.1 ± 3.3	0.087	0.15 (-0.04 – 0.35)
Highly supportive practice	Yes (≥20)	40 (19)	33 (16)	0.356	0.05 (-0.05 – 0.14)
**Work pace and EMR stress subscale (Q6-Q10)**	Mean ± SD	15.5 ± 3.1	15.0 ± 3.2	0.104	0.16 (-0.03 – 0.35)
Reasonable work pace and manageable EMR stress	Yes (≥20)	21 (10)	18 (9)	0.603	0.03 (-0.07 – 0.12)
**Sheehan Disability Scale (SDS)**					
**Total SDS score**	Mean ± SD	20.9 ± 6.0	22.8 ± 5.3	** <0.001**	0.34 (0.14 – 0.53)
Disrupted work	Mean ± SD	6.8 ± 2.3	7.4 ± 2.1	** 0.003**	0.27 (0.08 – 0.46)
Disrupted social life/leisure activities	Mean ± SD	7.8 ± 2.1	8.2 ± 1.9	** 0.047**	0.2 (0.01 – 0.39)
Disrupted family life/home responsibilities	Mean ± SD	6.2 ± 2.8	7.1 ± 2.5	** <0.001**	0.34 (0.15 – 0.53)

**Table 3:** Correlation matrix between the different scales, which is expressed as the Pearson correlation coefficient (*p-value*).

**Onset group (n=211)**			
	ISI score	Mini-Z summary score	SDS score
ISI score	1	-0.260 (***p<0.001***)	0.192 (***p=0.005***)
Mini-Z summary score	-0.260 (***p<0.001***)	1	0.006 (*p=0.932*)
SDS score	0.192 (***p=0.005***)	0.006 (*p=0.932*)	1
**One-year group (n=212)**			
ISI score	1	-0.370 (***p<0.001***)	0.343 (***p<0.001***)
Mini-Z summary score	-0.370 **(*p<0.001***)	1	-0.288 (***p<0.001***)
SDS score	0.343 (***p<0.001***)	-0.288 (***p<0.001***)	1

**Table 4:** Scores of insomnia, Mini-Z, and Sheehan disability scales for the onset sample participants.

**Characteristics**	**Total Scores**					
	**Insomnia Severity Index**		**Mini-Z survey**		**Sheehan Disability Scale**	
	**Mean (SD)**	***p-value***	**Mean ± SD**	***p-value***	**Mean ± SD**	***p-value***
**Age, y ®**						
23-27	8.76 (5.42)	0.172	31.04 (4.90)	** <0.001**	20.65 (6.13)	0.745
28-31	8.16 (5.77)		31.45 (5.49)		20.21 (5.24)	
32-39	9.46 (6.18)		31.12 (5.48)		21.31 (6.13)	
≥40	7.04 (5.41)		35.29 (5.31)		21.24 (6.49)	
**Gender**						
Male	8.35 (5.90)	0.844	32.38 (5.85)	0.355	20.68 (5.94)	0.506
Female	8.53 (5.35)		31.58 (4.62)		21.30 (6.02)	
**Marital Status**						
Never married	8.91 (5.90)	0.317	31.35 (5.17)	0.102	20.75 (5.20)	0.849
Married	8.09 (5.65)		32.64 (5.73)		20.91 (6.38)	
**Number of children in the family (among married ones)**						
Zero	7.77 (3.47)	** 0.019**	31.11 (5.57)	** 0.008**	20.08 (7.63)	0.199
1-2	9.84 (6.22)		32.00 (5.28)		20.86 (6.14)	
3-4	6.66 (5.26)		33.43 (5.38)		22.11 (5.71)	
>4	6.44 (4.69)		36.25 (6.05)		18.25 (7.66)	
**Living with the elderly (65 years or older)**						
No	8.44 (5.70)	0.904	32.03 (5.87)	0.708	19.92 (6.10)	** 0.010**
Yes	8.34 (5.83)		32.32 (5.14)		22.03 (5.58)	
**Occupation**						
Physician	8.36 (5.85)	0.857	32.09 (5.42)	0.734	20.93 (5.69)	0.722
Others	8.53 (5.41)		32.40 (6.05)		20.57 (6.85)	
**Working hours, weekly**						
<40	8.03 (5.26)	0.339	32.31 (5.99)	0.700	20.75 (5.94)	0.807
≥40	8.79 (6.22)		32.01 (5.07)		20.95 (6.00)	
**Monthly income, JD**						
<500	9.54 (6.96)	** 0.044**	31.63 (5.58)	** <0.001**	20.79 (4.81)	0.699
500-1000	9.04 (5.83)		31.00 (5.39)		20.47 (6.31)	
1000-2000	7.54 (4.67)		33.23 (5.71)		21.46 (4.97)	
>2000	6.39 (4.92)		35.20 (4.76)		21.61 (6.14)	
**COVID-19 testing**						
No	8.07 (5.63)	0.118	32.21 (5.58)	0.822	20.89 (6.09)	0.849
Yes	9.55 (6.06)		32.00 (5.50)		20.70 (5.52)	
**Direct contact with COVID-19 patients or samples**						
No	8.23 (5.60)	0.444	32.19 (5.73)	0.889	20.93 (5.94)	0.706
Yes	8.96 (6.25)		32.06 (4.93)		20.56 (6.05)	
**Perceived level of contact with COVID-19 patients**						
1 (Lowest)	6.45 (5.05)	0.070	32.92 (6.08)	** 0.001**	20.20 (6.16)	0.219
2	9.34 (6.11)		33.95 (5.22)		21.27 (5.89)	
3	8.68 (5.38)		29.98 (5.16)		21.23 (5.55)	
4	9.61 (6.05)		31.17 (4.95)		18.74 (6.80)	
5 (Highest)	7.87 (6.45)		32.80 (4.72)		22.87 (5.42)	
**Estimated number of COVID-19 patients that participants dealt with**						
Zero	8.22 (5.57)	0.308	32.20 (5.70)	0.690	20.93 (5.91)	0.900
1-49	9.65 (6.46)		31.65 (5.09)		20.47 (6.79)	
50-100	8.63 (7.03)		34.13 (5.62)		21.63 (4.93)	
>100	4.50 (1.73)		31.00 (2.45)		19.25 (2.22)	
**Receiving a special education to deal with COVID-19 patients**						
No	8.41 (5.65)	0.979	31.86 (5.58)	0.283	20.97 (6.02)	0.682
Yes	8.38 (5.96)		32.73 (5.48)		20.62 (5.87)	
**Participants’ evaluation of their institution's preparedness to deal with COVID-19 patients**						
Very bad	10.76 (8.10)	** 0.043**	25.00 (5.61)	** 0.001**	21.00 (7.00)	0.723
Bad	9.55 (5.74)		29.90 (5.58)		18.95 (7.73)	
Fair	7.59 (4.92)		32.26 (6.33)		21.62 (5.44)	
Good	8.69 (5.50)		31.61 (5.09)		20.99 (5.51)	
Very good	6.40 (5.73)		33.57 (4.49)		20.79 (5.79)	
Excellent	4.27 (4.58)		35.64 (6.30)		20.82 (7.85)	
**Perceived changes in work schedule and intensity due to the COVID-19 pandemic**						
No change	8.18 (5.24)	0.965	31.40 (4.12)	0.075	19.80 (7.58)	** 0.036**
A little	8.47 (5.79)		28.88 (6.16)		19.59 (7.75)	
Some	8.17 (5.68)		32.85 (5.78)		19.28 (5.43)	
Much	9.10 (5.72)		31.88 (5.71)		20.45 (5.56)	
Very much	9.12 (7.36)		32.99 (5.05)		22.60 (5.65)	
**Perceived changes in social life due to the COVID-19 pandemic**						
1 (No change)	7.06 (5.55)	0.286	27.18 (9.47)	** 0.004**	20.36 (8.31)	** <0.001**
2	8.48 (5.24)		31.30 (4.86)		16.12 (5.72)	
3	8.53 (5.80)		32.05 (5.15)		20.98 (5.24)	
4 (Extreme change)	10.82 (9.01)		33.33 (5.19)		22.73 (5.42)	

® Age was described as a categorical variable, with four groups divided by the interquartile ranges.

**Table 5:** Scores of insomnia, Mini-Z, and Sheehan disability scales for the one-year sample participants.

**Characteristics**	**Total Scores**					
	**Insomnia Severity Index**		**Mini-Z Survey**		**Sheehan Disability Scale**	
	**Mean (SD)**	***p-value***	**Mean ± SD**	***p-value***	**Mean ± SD**	***p-value***
**Age, y**						
23-27	8.16 (5.34)	0.086	29.86 (6.29)	** 0.023**	22.79 (6.16)	0.727
28-31	9.77 (7.32)		30.17 (5.27)		23.60 (4.88)	
32-39	9.85 (6.45)		31.03 (4.76)		22.93 (4.47)	
≥40	7.22 (5.90)		33.02 (6.14)		22.31 (5.81)	
**Gender**						
Male	8.64 (6.43)	0.942	31.00 (6.02)	0.630	22.71 (5.43)	0.784
Female	8.71 (6.08)		31.42 (5.17)		22.92 (4.90)	
**Marital Status**						
Never married	9.16 (6.24)	0.330	30.06 (5.39)	** 0.021**	22.64 (5.17)	0.755
Married	8.30 (6.35)		31.90 (5.92)		22.87 (5.35)	
**Number of children in the family (among married ones)**						
Zero	11.45 (6.31)	0.279	31.09 (6.36)	0.299	23.82 (5.40)	0.668
1-2	8.53 (6.36)		31.12 (5.51)		22.28 (5.01)	
3-4	7.43 (6.41)		32.45 (6.67)		23.15 (6.15)	
>4	7.36 (5.93)		34.21 (4.66)		23.79 (4.34)	
**Living with the elderly (65 years or older)**						
No	8.31 (6.41)	0.490	30.88 (5.27)	0.589	22.83 (4.78)	0.887
Yes	8.92 (6.24)		31.31 (6.12)		22.73 (5.61)	
**Occupation**						
Physician	8.81 (6.51)	0.325	30.80 (5.77)	** 0.050**	22.97 (5.39)	0.193
Others	7.81 (4.98)		32.97 (5.49)		21.66 (4.40)	
**Work hours, weekly**						
<40	8.18 (6.39)	0.258	32.34 (5.83)	** 0.001**	22.39 (5.30)	0.270
≥40	9.17 (6.21)		29.84 (5.43)		23.18 (5.22)	
**Monthly income, JD**						
<500	11.48 (5.79)	** 0.007**	28.71 (5.21)	** 0.004**	24.24 (5.03)	0.414
500-1000	9.29 (6.38)		30.35 (5.78)		22.58 (5.21)	
1000-2000	9.10 (7.12)		31.00 (5.12)		23.47 (4.37)	
>2000	6.65 (5.48)		33.08 (5.73)		22.27 (5.76)	
**COVID-19 testing**						
No	6.74 (6.54)	0.122	33.52 (5.70)	** 0.035**	21.13 (7.65)	0.270
Yes	8.89 (6.26)		30.84 (5.72)		22.97 (4.88)	
**COVID-19 infected**						
No	8.50 (6.28)	0.627	31.43 (5.74)	0.369	22.75 (5.38)	0.934
Yes	8.88 (6.37)		30.71 (5.80)		22.81 (5.13)	
**Direct contact with COVID-19 patients or samples**						
No	8.29 (6.21)	0.444	32.69 (5.01)	0.889	21.50 (5.47)	0.706
Yes	8.83 (6.41)		30.39 (5.97)		23.38 (5.07)	
**Perceived level of contact with COVID-19 patients**						
1 (Lowest)	5.69 (4.89)	0.055	36.69 (3.92)	** <0.001**	20.62 (4.09)	** 0.003**
2	7.55 (5.16)		32.27 (5.40)		20.14 (6.43)	
3	8.37 (6.71)		31.12 (5.61)		22.73 (4.90)	
4	8.24 (6.37)		31.59 (5.45)		22.44 (5.34)	
5 (Highest)	10.60 (6.18)		28.84 (5.80)		24.76 (4.66)	
**Estimated number of COVID-19 patients that participants dealt with**						
Zero	8.22 (6.12)	0.113	32.48 (4.95)	** 0**.** 015**	21.47 (5.44)	** 0.018**
1-49	8.00 (5.37)		31.30 (5.71)		22.72 (5.50)	
50-100	11.20 (7.82)		30.47 (6.29)		23.63 (5.21)	
>100	8.67 (6.88)		28.98 (6.26)		24.52 (3.97)	
**Receiving a special education to deal with COVID-19 patients**						
No	9.29 (6.82)	0.062	31.03 (5.26)	0.777	22.46 (5.62)	0.258
Yes	7.72 (5.36)		31.27 (6.47)		23.25 (4.66)	
**Participants’ evaluation of their institution's preparedness to deal with COVID-19 patients**						
Very bad	13.10 (5.55)	** 0.007**	23.30 (4.57)	** <0.001**	25.20 (7.76)	0.100
Bad	10.63 (7.26)		28.97 (5.20)		24.23 (4.63)	
Fair	8.85 (5.88)		30.15 (5.15)		23.08 (4.51)	
Good	8.34 (6.35)		32.51 (4.71)		21.41 (5.36)	
Very good	5.95 (5.40)		32.83 (7.00)		22.49 (5.82)	
Excellent	7.67 (4.59)		34.51 (5.98)		22.17 (5.00)	
**Perceived changes in work schedule and intensity due to the COVID-19 pandemic**						
No change	6.64 (4.87)	** <0.001**	34.19 (6.72)	** 0.009**	21.51 (4.69)	** 0.041**
A little	7.54 (4.93)		31.08 (5.68)		22.08 (3.38)	
Some	7.57 (6.31)		31.98 (5.26)		22.29 (5.24)	
Much	11.32 (6.79)		31.51 (5.96)		23.19 (3.90)	
Very much	11.50 (5.60)		29.02 (5.06)		24.54 (6.05)	
**Perceived changes in social life due to the COVID-19 pandemic**						
1 (No change)	6.76 (5.74)	** 0.003**	31.17 (4.49)	0.788	16.50 (8.17)	** <0.001**
2	9.50 (4.51)		31.67 (4.93)		20.53 (5.30)	
3	9.93 (6.68)		31.37 (5.75)		22.70 (4.51)	
4 (Extreme change)	10.19 (5.99)		30.64 (6.23)		24.28 (5.14)	

**Table 6:** Risk factors for clinical insomnia (Insomnia Severity Index of ≥15) among health care providers as identified by binary logistic regression analyses*.

**Variable**	**No. of Disease Cases/** **No. of Total Cases (%)**	**Adjusted OR**	**95% CI ** **(Min. **– **Max.)**	***p-value***
**Onset sample (n=211)**				
**Age, y**				
23-27	7/46 (15)	REF	REF	REF
28-31	7/57 (12)	0.851	0.260 – 2.783	0.790
32-39	11/59 (19)	2.727	0.744 – 9.994	0.130
≥40	6/49 (12)	6.919	1.097 – 43.621	** 0.040**
**Occupation**				
Physician	24/164 (15)	3.406	0.938 – 12.361	0.062
Others	7/47 (15)	REF	REF	REF
**Weekly working hours**				
<40	4/43 (9.3)	REF	REF	REF
≥40	27/168 (16.1)	2.084	0.645 – 6.728	0.220
**Monthly income, JD**				
<500	6/24 (25)	28.750	3.005 – 275.019	** 0.004**
500-1000	21/120 (18)	23.067	2.595 – 205.036	** 0.005**
1000-2000	2/26 (8)	4.803	0.494 – 46.710	0.176
>2000	2/41 (5)	REF	REF	REF
**Perceived level of contact with COVID-19 patients**				
1 (Lowest)	2/49 (4)	REF	REF	REF
2	12/62 (19)	5.811	1.150 – 29.374	** 0.033**
3	11/62 (18)	4.055	0.802 – 20.493	0.090
4	4/23 (17)	3.945	0.616 – 25.258	0.147
5 (Highest)	2/15 (13)	3.157	0.368 – 27.085	0.294
**One-year sample (n=212)**				
**Age, y**				
23-27	6/43 (14)	REF	REF	REF
28-31	12/51 (24)	4.219	1.115 – 15.955	** 0.034**
32-39	13/59 (22)	5.024	1.268 – 19.913	** 0.022**
≥40	8/59 (14)	3.184	0.652 – 15.541	0.152
**Marital status**				
Not married	19/89 (21)	2.340	0.861 – 6.362	0.096
Married	20/123 (16)	REF	REF	REF
**Receiving a special education to deal with COVID-19 patients**				
No	27/127 (21)	1.814	0.766 – 4.293	0.175
Yes	12/85 (14)	REF	REF	REF
**Perceived changes in social life due to the COVID-19 pandemic**				
1 (No change)	1/6 (17)	REF	REF	REF
2	8/36 (22)	1.036	0.084 – 12.808	0.978
3	9/87 (10)	0.233	0.019 – 2.866	0.255
4 (Extreme change)	21/83 (25)	0.628	0.052 – 7.565	0.714
**Other scales (continuous)**				
Mini-Z scale summary score	-	0.885	0.813 – 0.964	** 0.005**
Sheehan Disability Scale score	-	1.215	1.082 – 1.364	** 0.001**

* The age groups, gender, marital status, occupation, living with the elderly, weekly working hours categories, monthly income classifications, history of COVID-19 testing or infection, the participants’ assessment of their contacts with infected patients, getting COVID-19 related education, the participants’ evaluations of their institution’s readiness to deal with COVID-19 patients, their perception about workload and schedule changes, their perceived levels of change in their social life, and the total scores of the Mini-Z survey and SDS were included as independent explanatory variables in this model.

**Table 7:** Risk factors for burnout in health care providers based on binary logistic regression*.

**Variable**	**No. of disease cases/** **No. of total cases (%)**	**Adjusted OR**	**95% CI ** **(Min. **– **Max.)**	***p-value***
**Onset sample (n=211)**				
**Age, y**				
23-27	20/46 (43.5)	10.180	2.242 – 46.227	** 0.003**
28-31	12/57 (21.1)	1.923	0.453 – 8.157	0.375
32-39	13/59 (22.0)	2.118	0.589 – 7.610	0.250
≥40	6/49 (12.2)	REF	REF	REF
**Gender**				
Male	36/154 (23.4)	REF	REF	REF
Female	15/57 (26.3)	1.789	0.761 – 4.207	0.182
**Occupation**				
Physician	36/164 (22.0)	REF	REF	REF
Others	15/47 (31.9)	2.422	0.828 – 7.083	0.106
**Monthly income, JD**				
<500	11/24 (45.8)	8.815	1.39 – 55.871	** 0.021**
500-1000	34/120 (28.3)	1.443	0.283 – 7.346	0.659
1000-2000	3/26 (11.5)	1.375	0.196 – 9.640	0.748
>2000	3/41 (7.3)	REF	REF	REF
**Perceived changes in social life due to the COVID-19 pandemic**				
1 (No change)	1/11 (9.1)	REF	REF	REF
2	5/33 (15.2)	0.360	0.042 – 3.063	0.350
3	22/87 (25.3)	3.608	0.748 – 17.394	0.110
4 (Extreme change)	23/80 (28.8)	6.225	1.252 – 30.946	** 0.025**
**Other scales (continuous)**				
Insomnia Severity Index (ISI)	-	1.118	1.053 – 1.187	** <0.001**
Sheehan Disability Scale score	-	1.102	1.028 – 1.180	** 0.006**
**One-year sample (n=212)**				
**Age, y**				
23-27	23/40 (57.5)	3.927	1.185 – 13.016	** 0.025**
28-31	21/49 (42.9)	2.669	0.724 – 9.840	0.140
32-39	19/59 (32.2)	1.486	0.470 – 4.701	0.500
≥40	12/64 (18.8)	REF	REF	REF
**Gender**				
Male	46/147 (31.3)	REF	REF	REF
Female	29/65 (44.6)	1.908	0.885 – 4.109	0.099
**Monthly income, JD**				
<500	13/21 (61.9)	4.707	1.132 – 19.568	** 0.033**
500-1000	38/95 (40.0)	1.682	0.550 – 5.141	0.362
1000-2000	11/30 (36.7)	1.754	0.491 – 6.266	0.387
>2000	13/66 (19.7)	REF	REF	REF
**Working hours, weekly**				
<40	15/59 (25.4)	REF	REF	REF
≥40	60/153 (39.2)	3.585	1.418 – 9.066	** 0.007**
**Participants’ evaluation of their institution's preparedness to deal with COVID-19 patients**				
Very bad	7/10 (70.0)	42.333	1.595 – 123.476	** 0.025**
Bad	18/35 (51.4)	4.598	0.435 – 48.577	0.205
Fair	25/65 (38.5)	1.845	0.182 – 18.701	0.604
Good	12/58 (20.7)	0.699	0.066 – 7.367	0.766
Very good	10/37 (27.0)	0.962	0.079 – 11.697	0.976
Excellent	3/7 (42.9)	REF	REF	REF
**Perceived changes in work schedule and intensity due to the COVID-19 pandemic**				
No change	1/14 (7.1)	REF	REF	REF
A little	5/12 (41.7)	5.610	0.228 – 138.244	0.292
Some	10/45 (22.2)	6.796	0.412 – 112.012	0.180
Much	31/82 (37.8)	23.004	1.497 – 353.495	** 0.024**
Very much	28/59 (47.5)	23.578	1.531 – 363.230	** 0.024**
**Other scales (continuous)**				
Insomnia Severity Index score	-	1.113	1.038 – 1.192	** 0.003**
Sheehan Disability Scale score	-	1.109	1.025 – 1.201	** 0.010**

* The age groups, gender, marital status, occupation, living with the elderly, weekly working hours categories, monthly income classifications, history of COVID-19 testing or infection, the participants’ assessment of their contacts with infected patients, getting COVID-19 related education, the participants’ evaluations of their institution’s readiness to deal with COVID-19 patients, their perception about workload and schedule changes, their perceived levels of change in their social life, and the total scores of the ISI and SDS were included as independent explanatory variables in this model.

## Data Availability

The datasets generated and analyzed during the current study are available from the corresponding author [A.Y].

## LIST OF ABBREVIATIONS

HCP: = Health Care Providers
SD: = Standard Deviation
ISI: = Insomnia Severity Index
